# Surviving Pulmonary Tuberculosis: Navigating the Long Term Respiratory Effects

**DOI:** 10.7759/cureus.38811

**Published:** 2023-05-10

**Authors:** Arpit Bansal, Vishnu R Yanamaladoddi, Sai Suseel Sarvepalli, Shree Laya Vemula, Saikumar Aramadaka, Raam Mannam, Rajagopal Sankara Narayanan

**Affiliations:** 1 Internal Medicine, Narayana Medical College, Nellore, IND; 2 General Surgery, Narayana Medical College, Nellore, IND; 3 Internal Medicine, ACSR Government Medical College, Nellore, IND

**Keywords:** lung damage caused by tuberculosis, pulmonary function, pulmonary function after tuberculosis, lung damage, obstructive disease after tuberculosis, restrictive disease after tuberculosis, chronic respiratory disorders after tuberculosis, long term effects of tuberculosis, tuberculosis

## Abstract

*Tuberculosis* is a transmissible disease caused by the bacteria Mycobacterium tuberculosis, which is a cause of significant morbidity and mortality all over the world. Tuberculosis has a number of risk factors, such as living in a developing country, poor ventilation, smoking, male sex, etc., which not only increase the chance of infection but may be independent factors for impairment in pulmonary function as well. In this review article, we have compiled several studies to learn how tuberculosis causes impaired lung function and further explored the long-term effects of tuberculosis on the same. We studied tuberculosis's effect on the lungs even after appropriate treatment and its relationship with obstructive and restrictive lung disorders. A significant relationship exists between chronic respiratory disorders and tuberculosis even after treatment; hence, we believe prevention is far superior to cure.

## Introduction and background

Tuberculosis (TB) is a disease known to humans since ancient times [1]. There are several documented sources of TB descriptions in works from the Ancient Period, middle ages, and the Renaissance. The Old Testament (3700-3300 BC) has verses in which the Lord threatens with a disease called Consumption which is believed to be TB [2,3]. Ancient Indian texts, The Rigvedas, also describe a disease that loosely translates to "Consumption". Hippocrates (460-375 BC) believed TB to be hereditary and coined the term phthisis for the condition. Aretaeus of Cappadocia gave a very accurate picture of the symptoms of pulmonary TB in his book "FIRST BOOK OF CHRONIC DISEASES" in 200 BC [4]. It wasn't until 1882 that Robert Koch identified and isolated Mycobacterium tuberculosis as the causative agent of tuberculosis and gave his famous postulates to probe causation [5].

Tuberculosis is caused by Mycobacterium tuberculosis (MTB), an obligate aerobe. It has a thick waxy cell wall that allows it to escape the host's immune response. It is well established that the transmission of TB is airborne. Thus, a majority of the cases are of pulmonary TB (>85%) [6]. However, later on, it can spread to almost any part of the body via hematogenous, lymphatic, or direct pathways. The pathogenesis is initiated through the deposition of the bacilli in the distal lung. The initial infection occurs in the alveoli and later develops into a Ghon Focus, which can spread to regional lymph nodes, then known as the Ghon Complex, which can then progress into an open or closed cavity with a fibrous rim around an inner pool of liquefaction and caseous necrosis. This can further progress to lung fibrosis and destruction [7].

According to the WHO, TB is the second leading cause of death from a single infectious disease agent after Covid [8]. Scientists used Gaussian process regression to estimate that 1.7 billion were latently infected with tuberculosis in 2014 [9]. Furthermore, out of these individuals, 56 million people were at an increased risk of developing active disease, of which 11% carried a resistant strain of MTB. According to the Centers for Disease Control and Prevention (CDC), the incidence of TB infection in the US has been declining, and 2/3rds of new cases were reported in foreign-born individuals. The incidence rate in the United States in 2018 was only 2.8 cases per 100,000 population, whereas the worldwide incidence was 132 cases per 100,000 population in the same period [8].

**Figure 1:**
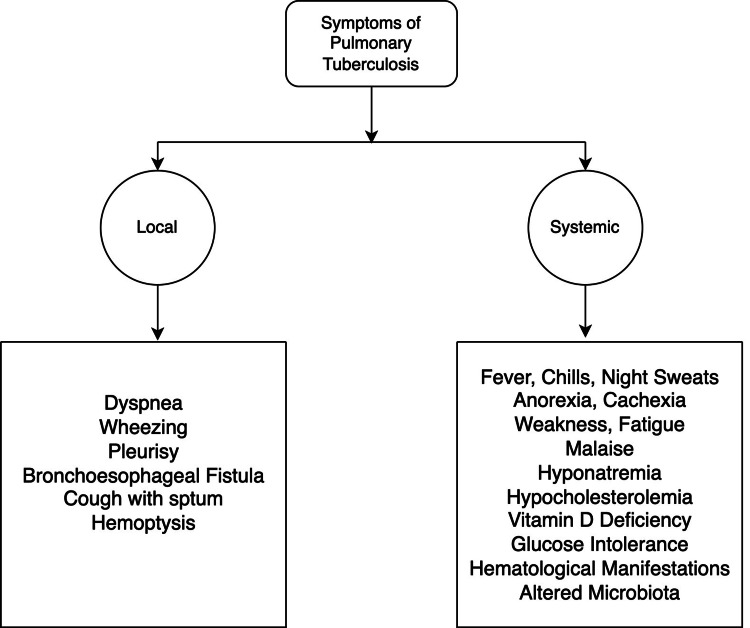
Symptoms of Tuberculosis. Image credits - Dr. Arpit Bansal

Tuberculosis has a number of known risk factors, including but not limited to young age, male gender, poor ventilation, living in a developing country, being a health care worker, exposure to contaminated air, smoking, and family history of TB [10]. It has also been proven that HIV infection and, thus, AIDS are prominent risk factors for contracting TB [11]. In active TB, cytokines, transcription factors, and chemokines increase, leading to lung damage and necrosis [12]. This mediates granuloma production directly or via tissue-degrading enzymes. Granuloma rupture leads to the introduction of bacilli into the pleural space and interaction with T cells which causes a hypersensitivity reaction to mycobacterial proteins and a marked increase in fluid in the pleural space. Granulomas may also lead to localized or widespread tissue fibrosis. [13]. The pleural effusion further presents with features of lung compression such as dyspnea, pleurisy, cough, and severe inflammation [14]. Furthermore, systemic symptoms such as fever, wasting, and "night sweats" are the hallmarks of tuberculosis, even leading to TB being called "the wasting disease" (Figure 1) [15]. Other symptomatic complications include hyponatremia, hypocholesterolemia, vitamin D deficiency, glucose intolerance, and altered microbiota [16].

Traditionally, TB is diagnosed using a number of techniques in conjunction with each other- clinical suspicion, chest X-ray, microscopy, and solid media culture [17]. Newer diagnostic techniques such as Nucleic acid amplification tests (NAATs), interferon-gamma release assays (IGRAs), and liquid culture systems have emerged, but amongst all the techniques, staining for acid-fast bacilli (AFB) has been one of the most effective and easy-to-use methods for TB diagnosis. The sensitivity of staining for AFB for pulmonary TB is ~60%, though it may differ with the course of the disease at which the sample was taken [18]. Thus, the WHO recommends the collection of two same-day "spot" collections to evaluate TB [19]. Tuberculosis is treated with the goals of reducing actively growing bacteria, killing all existing bacteria, and preventing the development of drug resistance all the while [20]. Keeping these goals in mind, pulmonary TB is treated with a combination of drugs and in different phases, depending on the drug sensitivity of the organism.

But a 2020 meta-analysis has found that even with treatment, there is increased mortality among those people compared to controls [21]. People treated for tuberculosis still have pulmonary complications despite successful treatment. In the USA, lung impairment was a common feature of people treated successfully for tuberculosis [22,23]. According to another meta-analysis, pulmonary tuberculosis is an independent risk factor for COPD and spirometric restriction [23].

In this review, we will discuss the association between tuberculosis and chronic respiratory diseases and the impact of TB on people's long-term health. We will explore these questions in further detail in our review.

## Review

Mediators for lung damage

Small aerosol droplets containing M. tuberculosis that are expectorated by coughing, sneezing, talking, or singing can spread TB from a patient with active disease to an uninfected person. These droplets pass through the respiratory system, where mucus-secreting goblet cells capture the majority of the bacilli. However, in certain situations, these droplets can slip through this first-line mucociliary defense system and enter the upper aerated regions of the lungs. This is when the host's innate immunity comes into play, and alveolar macrophages engulf the bacteria and combat them by secreting cytokines and enzymes, including tumor necrosis factor-alpha (TNF α) and interferon-gamma (IFNγ). This signals T lymphocytes to reach the site of infection, initiating a cell-mediated immune response, which often results in the formation of a granuloma, which is one of the most defining features of pulmonary TB. Over time, macrophages differentiate, resulting in a stratified structure with a layer of lymphocytes surrounding a macrophage-rich layer. This cannot eliminate the bacilli but suppresses further activity in an immunocompetent individual; this state is known as latent TB. Some granulomas may remain inactive for life, but in others, structural integrity may be lost, allowing for the formation of a cavity in the airway wall, contributing to lung damage in TB [6]. Granulomas may undergo aberrant repair leading to localized or widespread tissue fibrosis, and have varied resolution trajectories throughout the illness or treatment [13]. Therefore, it is possible that the immune responses that drive inflammation, cavitation, and fibrosis play a role in the different ways the lung heals and later manifests as lung injury [12].

**Figure 2:**
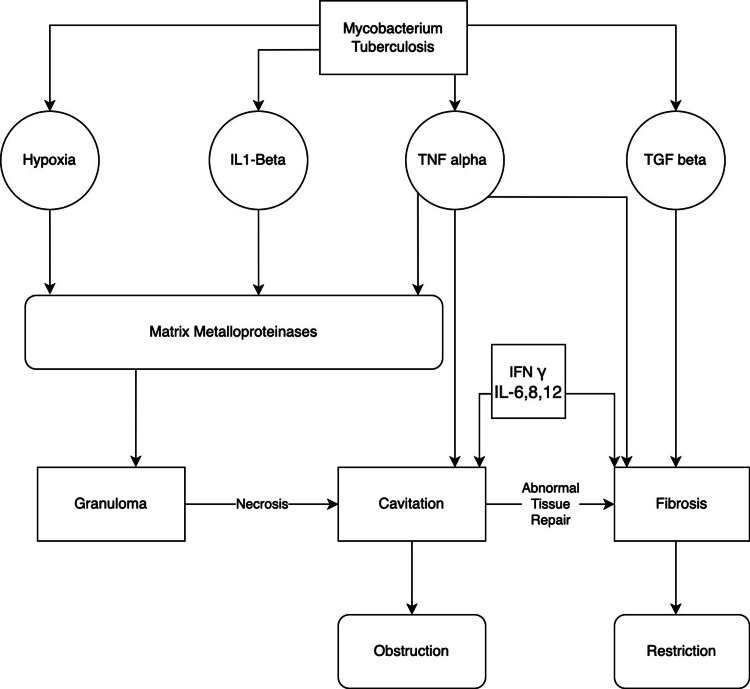
Mechanism of Lung Injury in Tuberculosis Image credits - Dr. Arpit Bansal TNF - Tumor Necrosis Factor; IL - Interleukin; TGF - Transforming Growth Factor; IFN - Interferon

A family of powerful proteases known as matrix metalloproteinases (MMPs) is thought to be the primary cause of TB-related lung damage as they have the ability to break down extracellular matrix components [14]. In TB, MMPs encourage various stages of lung remodeling [15]. According to recent imaging studies, human TB lesions are profoundly hypoxic. Nuclear factor (NF)-κB activation and hypoxia-inducible factor caused MMP-1 to be upregulated in MTB-infected cells when hypoxia was replicated in in-vitro culture conditions [16]. MTB was found to cause an imbalance in MMP-1 and its particular inhibitor, TIMP-3, in a rabbit model of cavitary TB illness, which was linked to the development of consolidated lung areas into cavities [17]. Therefore, unchecked MMP expression and activity may result in tissue damage, which eventually causes lung injury (Figure 2) [12].

TNF α is a key regulator of host immune responses to TB with a number of effects [18]. TNF- α's key host-protective function is intracellular pathogen clearance via the activation of macrophages [18]. Low TNF- α levels could be problematic as they have been linked to ineffective macrophage activation and decreased microbicidal activity. Excessive inflammation and necrosis may result from unchecked MTB multiplication [19]. In addition to TNF- α, increased Interleukin-6, Interleukin-8, and Interleukin-12(IL-6,8 and 12) levels have been linked to the formation of cavities, bronchial wall thickening, and fibrotic bands in individuals with active tuberculosis [20]. In another study, the improvement in chest radiographs after 2 or 6 months of TB treatment was used to categorize patients as early or late responders, respectively. In contrast to early responders, late responders exhibited higher levels of IL-1, TNF, and IFN, suggesting that these cytokines may have contributed to lung tissue injury [21]. Lung remodeling may continue during and after TB treatment as inflammatory cytokine levels are highly dynamic soon after its initiation [22,23].

During TB disease and therapy, excessive collagen deposition and fibrotic scarring may develop [13,24]. TGF β is considered the principle mediator of fibrogenesis [25]. Prior to and throughout TB therapy, lung lesions with activated TGF-signaling pathways had higher collagen levels [24]. Idiopathic pulmonary fibrosis (IPF) is a deadly lung condition that progresses and is characterized by restrictive ventilatory abnormalities. IL-1β has been related to fibrosis in patients with IPF [26]. Patients with a history of TB may experience restrictive ventilatory abnormalities due to TNF- α, TGF- β, and IL-1-mediated fibrogenesis [12].

The initiation, development, progression, and eventual lung destruction in TB have all been linked to various cell types. Alveolar macrophages found in the lungs are likely to be the first cell type to contract MTB during primary infection. Upon activation, these cells release inflammatory cytokines and chemokines that attract innate and adaptive immune cells to the infection site [13,27,28]. Caseation and cavitation are likely caused as a result of the dysregulation of immunological responses [29,30]. When responding to TB, CD4 T-cells develop defensive responses, but if their responses are left unregulated, these cells will continue to cause tissue damage [31,32]. It's possible that TB-specific CD4 T cells that release TNF and IFN also activate other downstream pathways and effectors like MMPs. These reactions could result in tissue damage, excessive inflammation, and consequent lung impairment [12].

Following TB, the lungs may experience gas exchange impairment, airflow blockage, and obstructive and/or restrictive ventilatory abnormalities [33-36]. 

Tuberculosis and airflow obstruction

Spirometry is a diagnostic tool that measures the flow and quantity of air expelled by the lungs and can be used to identify airflow abnormalities [37]. Standardized recommendations for performing and interpreting these tests are provided by the American Thoracic Society and the European Respiratory Society [38-40]. Inflammation-induced airway narrowing likely causes airflow obstruction, which is characterized by a reduced ability to completely remove air from the lungs. Reduced ability to completely remove air from the lungs is linked to airflow obstruction, likely caused by inflammation-induced airway narrowing [12]. Measuring the forced expiratory volume in 1 second is a standard approach to determining the severity of airway obstruction (FEV1) [39]. FEV1 is expressed as a percentage of expected normal and absolute volume, with an FEV1 drop of 100 mL drop from baseline being considered clinically significant [41]. In 2009, Maguire et al. conducted a prospective cohort study with patients with smear-positive pulmonary TB in Papua, Indonesia. FEV1 in one second was found to be less than 60% of the predicted value in 39% of the subjects, signifying pulmonary impairment. [42]. Manji et al. conducted a cross-sectional study in 2016, in which the results pointed to a significant relationship between pulmonary tuberculosis and abnormal lung function, with 42% of patients having an obstructive pattern and 19% of patients having a mixed obstructive and restrictive pattern [43]. In another study conducted by Lee et al. in 2011 in South Korea, it was concluded that prior TB was a risk factor for obstructive lung disease, and even a small lesion from tuberculosis was a strong risk factor for obstructive lung disease [44]. In a systematic review conducted by Byrne et al. in 2014, there was a significant association between tuberculosis and COPD among adults over 40 years of age [34]. Pasipanodya et al., in 2007, conducted a case-control study in which it was found that patients with pulmonary tuberculosis receiving treatment had significantly lower values for FVC, FEV1, FEV1/FVC ratio, and the mid-expiratory phase of forced expiratory flow than those patients in the comparison group [45]. In another cross-sectional study conducted by Willcox et al. in 1989, the results indicated an inverse relationship between the extent of disease and the FEV1 after one second in the subjects [35]. In a multicenter retrospective study conducted in South Korea by Rhee et al. in 2013, patients had declining FEV1 and decreased lung function with exacerbation [46]. Hence it can be stated that a decrease in FEV1 is seen both during and following TB treatment(Table 1) [33,34,42,45-47].

Bronchiectasis

With recurrent episodes of purulent sputum production, hemoptysis, and occasionally the development of pneumonia, bronchiectasis is a chronic deformation of the airways that predisposes to lifelong illness [48]. It is known that bronchiectasis is a pulmonary tuberculosis sequela that can continue or worsen even after TB therapy is finished [49,50]. In post-mortem autopsy investigations of TB patients conducted by Jones et al. in 1950, bronchiectasis was discovered in 19-65% of cases [51]. In another study conducted by Salkin et al. in 1950, tuberculosis was found to be the cause of bronchiectasis in a significant number of subjects studied [52]. It is concerning to see that in a recent cross-sectional and descriptive study performed by Capone et al. in 2017, 86% of patients developed cylindrical bronchiectasis on chest computed tomography (CT) at 6 months after TB treatment [53]. They concluded that it was most likely that late diagnosis was to blame for the high rate of persistent changes following treatment and that CT findings of tuberculosis persist even after treatment. According to five CT studies evaluated in a systematic review by Meghji et al. in 2016, the prevalence of bronchiectasis following TB ranged from 35-86% [54]. They concluded that long-term lung damage was common after pulmonary tuberculosis, but there was still a need to conduct more studies to ascertain the development of this damage and its associated morbidity over time. In addition, Zhou et al. conducted a population-based study of more than 10,000 adults in China in 2013, concluding that those with a history of tuberculosis had a three times greater risk of receiving a bronchiectasis diagnosis than those without a history of the disease [55]. According to a comparative study conducted by Jin et al. in 2018, patients with prior pulmonary tuberculosis had higher rates of bronchiectasis, which was more prevalent in lungs with tuberculosis lesions, and a larger percentage of more severe bronchiectasis than those without prior pulmonary tuberculosis [56]. They had longer histories of dyspnea, more exacerbations, and more positive bacteria cultures than people without a history of pulmonary tuberculosis. Bronchiectasis as a complication was found to be more common in patients with cavitary lesions than with non-cavitary disease (64% vs. 11 %; p<0.05) [36]. Table 1 summarizes the various studies which showed an association between tuberculosis and chronic respiratory disorders.

**Table 1:** Studies exploring the association between tuberculosis and chronic respiratory diseases. TB - Tuberculosis; COPD - Chronic Obstructive Pulmonary Disease; FEV1 - Forced expiratory volume in the first second; FVC - Forced Vital Capacity; RV - Residual Volume; pred - predicted; TLC - Total Lung Capacity

First Author	Type of Study	Setting	Exposure	Outcome	Association/Finding
Plit et al. [33] (1998)	Prospective Cohort	South Africa	TB Treatment	Lung function at the end of TB treatment	The lung function of 54% of patients improved, 28% of patients had obstruction, and 24% of patients had restricted airflow.
Byrne et al. [34] (2015)	Systematic review and meta-analysis	Multiple Countries	History of TB	COPD	In persons over 40, a history of TB was strongly linked to COPD.
Pasipanodya et al. [45] (2007)	Case-Control	USA	Latent VS Active cases	Airway obstruction defined as FEV1/FVC <70% pred and FVC >80% pred	Compared to controls with latent TB, TB patients on anti-TB therapy have significantly increased risks of pulmonary impairment.
Maguire et al. [42] (2009)	Prospective Cohort	Indonesia	TB treatment	Lung function throughout TB treatment	Although lung function improved during the TB treatment, 25% of the patients still had moderate-to-severe lung impairment (FEV1 60%) when the treatment was over.
Willcox et al. [35] (1989)	Cross-sectional	South Africa	History of TB	Airflow obstruction defined as RV >120% pred and/or FEV1/FVC ratio <70% pred with TLC >80% of pred	68% of patients have obstruction, 20% obstruction with some restriction, and 17% non-obstructive reduction in lung volume
Akkara et al. [57] (2013)	Cross-sectional	India	TB Treatment	Airflow obstruction measured by FEV1 and FVC	86.8% of patients experience airflow obstruction.

Restrictive ventilatory defects

Restricted airflow is another issue that may affect patients after treatment of tuberculosis [33,57]. Reducing forced vital capacity (FVC), an increase, or even preservation of the FEV1/FVC ratio can all signify restriction [40]. In a cross-sectional study conducted in India by Akkara et al. in 2013, it was found that the majority of the patients showed a mixed pattern of disease [57]. They concluded that people suffering from pulmonary tuberculosis not only need timely diagnosis and management but also long-term follow-up for the diagnosis and treatment of any functional impairment as a consequence of pulmonary tuberculosis. In another cross-sectional study conducted by Manji et al. in 2016, there was a significant relationship between pulmonary tuberculosis and abnormal lung function, with 13% of patients having a restrictive pattern and 42% of patients having an obstructive pattern, while 19% showcasing a mixed pattern of abnormal lung function [43]. Their findings also suggest lung damage after pulmonary tuberculosis is associated with the male sex, age older than 40 years, recurrent pulmonary tuberculosis, and a negative HIV status. In a study conducted by Plit et al. in 1989, 57% and 24% of patients, respectively, had restrictions found at baseline and upon the conclusion of treatment [33]. A review of population-based and observational studies undertaken in South Africa by Ehrlich et al. found that mixed patterns of airflow obstruction/restrictive ventilatory abnormalities were the most prevalent type of lung dysfunction, even though airflow obstruction in TB has garnered the majority of attention [58]. In a review conducted by Ravimohan et al. in 2017, they stated that airflow restriction in TB patients might be explained by structural abnormalities in the lung brought on by aberrant lung tissue regeneration [12].

Impaired diffusion capacity

According to a systematic review by Malmberg et al. in 1966, early in the TB disease, there is evidence of decreased gas exchange as well [59]. A comparative study conducted by Kim et al. in South Korea in 2004 showed that in post-tuberculosis emphysema, diffusing capacity and right ventricular ejection fraction were lowered [60]. In a prospective study conducted by Long et al. in 1998, it was concluded that tuberculosis causes a reduction in both ventilation and perfusion [36]. This explains the minimal functional impairment witnessed in these patients despite extensive lung damage. Even when the diffusing capacity for carbon monoxide(DLCO) improves with treatment, some people may have chronic hypoxemia due to poor diffusion and ventilation/perfusion mismatch [36,60].

Limitations

This review is limited by the studies conducted on this topic and the small sample sizes of the studies conducted. Also, in many cases, we cannot say if independent factors may be causing pulmonary impairment after the treatment of tuberculosis. The article concentrates exclusively on sequelae of pulmonary TB and does not discuss any extrapulmonary sequelae of TB.

## Conclusions

As evident from the studies reviewed in this article, despite adequate treatment for tuberculosis, it does not lead to full recovery of pulmonary function. There is definite morbidity associated with tuberculosis, even with adequate treatment, which is not associated with the expected complications of the disease. We spoke of the deleterious effects of the disease on the lungs, resulting in obstructive diseases, restrictive diseases, impaired exchange of gases, and a combination of the above. We strongly believe prevention is better than cure, and this article fully supports this. Early detection and treatment may mitigate some harmful long-term effects on the lungs. We believe there is a definite need for better education to prevent tuberculosis. Studies need to be conducted to establish if early treatment may mitigate some of the long-term effects of tuberculosis on pulmonary function. Furthermore, there is a need for more in-depth research into the relationship between tuberculosis and chronic respiratory diseases to fully understand its effects on lung conditions to reduce associated morbidity and improve patients' quality of life.
